# Systematic Analysis of an Immune-Related Gene Signature for Predicting Prognosis and Immune Characteristics in Primary Lower Grade Glioma

**DOI:** 10.1155/bmri/6180391

**Published:** 2025-08-12

**Authors:** Liubing Hou, Lei Tian, Jiayuan Li, Zizhou Zhang, Xuetao Han, Huandi Zhou, Xiaoying Xue

**Affiliations:** ^1^Department of Radiotherapy, The Second Hospital of Hebei Medical University, Shijiazhuang, Hebei, China; ^2^Department of Central Laboratory, The Second Hospital of Hebei Medical University, Shijiazhuang, Hebei, China

**Keywords:** immune checkpoints, immune-related gene, lower grade glioma, prognosis model, ssGSEA, tumor immune microenvironment

## Abstract

**Background:** Immune-related genes (IRGs) have been increasingly recognized as critical determinants in the multistage processes of cancer development and progression. However, the functional roles of IRGs in the incidence and progression of LGG remain to be studied. This study is aimed at establishing a robust IRGs signature through systematic bioinformatics analysis, followed by an in-depth investigation of the molecular mechanisms underlying its functional roles. A key objective is to dissect the intricate interplay between IRGs expression patterns and the composition/functional orientation of tumor-infiltrating immune cells inside the tumor microenvironment. Furthermore, our findings are aimed at providing novel evidence to facilitate molecular diagnosis and advance immunotherapeutic strategies for LGG.

**Methods:** RNA sequencing datasets, accompanied by detailed and pertinent clinical information pertinent to LGG, were meticulously retrieved from databases including TCGA and CGGA. To measure the levels of immune cell distribution across the specimens, we employed the sophisticated ssGSEA, which incorporated 29 immune infiltration-related information, enabling stratification of cases into immunity-low (immunity_L) and immunity-high (immunity_H) clusters. This classification provided crucial insights for understanding the immune landscape of LGG and its potential clinical implications. To further investigate, we identified differentially expressed IRGs by intersecting the list of DEGs with the IRGs curated from the ImmPort website. Subsequent feature selection employed Cox proportional hazards regression and LASSO regression to derive a prognostic IRGs signature in the TCGA cohort. This model facilitated risk stratification of patients into low-risk and high-risk subgroups. The established signature's predictive efficacy was rigorously validated in the CGGA cohort through comprehensive analytical approaches. This included Kaplan–Meier survival analysis for prognostic stratification, time-dependent receiver operating characteristic (ROC) curve construction for quantifying predictive accuracy, principal component analysis (PCA) for visualizing sample distribution patterns, and subgroup stratification to assess consistency across clinical variables. Additionally, ssGSEA was utilized to profile the TME, and correlation analyses were performed between the IRG-derived risk score and immune checkpoint expression levels.

**Results:** Finally, we selected CXCL10, ICAM1, IL18, ITGAL, SOCS3, and TLR3 to establish a six-gene IRGs signature for LGG. Based on this feature, we divided patients into low- and high-risk subgroups and found that high-risk patients consistently exhibited shorter OS. Notably, the risk score based on this signature emerged as an independent predictor of OS. TME analysis showed more immune infiltration in the high-risk subgroup. Correlation analysis further revealed a strong positive association between the risk score and TIM3 expression in both TCGA and CGGA datasets, with significantly higher TIM3 expression in the high-risk group. Individual analyses of these six genes revealed that elevated expression levels of CXCL10, ICAM1, IL18, ITGAL, SOCS3, and TLR3 were detected in tumor tissues compared to adjacent normal tissues. Notably, overexpression of these immunoregulatory genes demonstrated a significant correlation with unfavorable clinical outcomes in patients' survival analysis. Similar results were obtained in the tissue samples validation conducted at our center.

**Conclusion:** The study developed an innovative signature encompassing six IRGs that accurately predicts prognosis, offers potential for identifying prognostic biomarkers, and may guide individualized immunotherapy for LGG.

## 1. Introduction

Gliomas constitute the most prevalent primary malignancies of the central nervous system (CNS), characterized by an extremely poor prognosis [1]. Infiltrating gliomas exhibiting differentiation are collectively classified as lower grade glioma (LGG), corresponding to histopathological Grades II and III. LGG arises most commonly in the adult cerebral hemispheres, including astrocytoma, oligodendroglioma, and oligoastrocytoma. The highly aggressive and invasive nature of gliomas poses significant challenges for complete elimination using current therapeutic strategies, including surgery, radiotherapy, and chemotherapy. Residual tumor cells often lead to recurrence and malignant progression, with some gliomas having a tendency to progress into glioblastoma (WHO Grade IV glioma) within a short span of a few months, resulting in a poor prognosis [2]. Consequently, there is an urgent need to identify innovative treatment approaches for glioma. Prognostic markers, as crucial tools predicting patient survival, disease recurrence risk, and treatment response, serve as a critical therapeutic modality in contemporary oncological management. For LGG, the identification of immune cell markers or IRGs with prognostic value not only provides doctors with more accurate prognostic information to guide treatment decisions but also monitors disease progression, enabling timely adjustments to treatment regimens. Furthermore, in-depth research into these prognostic markers aids in elucidating the pathogenesis of LGG, providing a theoretical foundation and experimental basis for the development of novel therapeutic approaches and medications. This study, based on the aforementioned background, aims to identify IRG signatures associated with LGG prognosis using bioinformatics methods, offering new insights into precision therapy and prognostic assessment for LGG.

The tumor microenvironment (TME) represents a dynamic ecosystem comprising heterogeneous stromal cell populations, diverse immune cell subsets, complex extracellular matrix components, and an array of bioactive soluble factors. These constituents collectively orchestrate intricate cross-talk that critically influences tumor initiation, growth, and metastatic dissemination [3, 4]. Detailed investigations into the TME have revealed its significance in tumor advancement, immune escape, and the response to immunotherapy. Recent studies have demonstrated that immunotherapies targeting immune cells or immune checkpoints can enhance survival outcomes in patients with colorectal cancer [5], lung cancer [6] and various other tumor types, establishing them as promising therapeutic modalities in cancer treatment [7]. However, it is noteworthy that specific immunotherapy clinical trials—particularly those evaluating PD-1 checkpoint inhibitors in glioma management—have not demonstrated the expected survival advantages [8]. This outcome underscores the critical need for comprehensive molecular dissection of the TME to uncover novel therapeutic targets and develop innovative intervention strategies capable of overcoming current limitations in immunotherapy effectiveness [9].

In the comprehensive study concentrated on LGG pathogenesis, we systematically integrated multiomics data from authoritative public repositories to construct an IRGs prognostic model. The genomic landscape analysis incorporated RNA-sequencing data and microarray profiles from The Cancer Genome Atlas (TCGA) database, complemented by the Chinese Glioma Genome Atlas (CGGA) to enhance population diversity. Normal brain tissue controls were rigorously matched using GTEx database samples, ensuring appropriate baseline comparisons. Through the application of univariate regression analysis, we detected survival-related genetic markers. Subsequently, we constructed an IRGs prognostic model through the implementation of least absolute shrinkage and selection operator (LASSO) regression methodology combined with Cox proportional hazards analysis. The IRGs signature, comprising CXCL10, ICAM1, IL18, ITGAL, SOCS3, and TLR3, exhibited a remarkable ability to meticulously predict LGG patients' prognosis. Furthermore, our analysis uncovered substantial associations between the IRG-based prognostic model and immune cell infiltration degree in the LGG TME. Our results demonstrated the intimate link between the IRGs signature and prognosis, offering reliable insights into the microenvironment of primary LGG samples and paving the way for further elucidation of this complex disease.

## 2. Materials and Methods

### 2.1. Data Collection

For this investigation, we obtained multiomics data comprising transcriptomic profiles and corresponding clinical annotations for 538 histologically confirmed LGG cases from the TCGA data portal (https://portal.gdc.cancer.gov/). Following the exclusion of recurrent glioma, a final cohort comprising 511 samples was established, among which 506 contained complete survival data. To establish a normal tissue reference, RNA sequencing data from 347 nontumor brain specimens was obtained from the GTEx database (https://xenabrowser.net/datapages/). The “normalizeBetweenArrays” function within the “limma” package in R was utilized to achieve quantile normalization and integration of both TCGA and GTEx datasets.

To strengthen the analytical robustness of our study, we integrated mRNAseq_693 and mRNAseq_325 along with their corresponding clinical annotations from the CGGA database (http://www.cgga.org.cn/) This multicohort integration generated a comprehensive dataset containing 1018 glioma samples, from which 592 histologically verified LGG cases with complete clinical and molecular characterization were selected for model validation. IRGs were systematically compiled from the Immunology Database and Analysis Portal (ImmPort) (https://www.immport.org/home) to support our investigation of immune-related molecular signatures. The list comprised 1793 IRGs after removal of duplicates. The methodologies employed in this study were aligned with those described by Wang et al. in 2021 [10].

### 2.2. Evaluation of the TME Through Single-Sample Gene Set Enrichment Analysis (ssGSEA) and Enrichment Analysis of GSEA

To comprehensively characterize the immune landscape of LGG, we implemented the ssGSEA algorithm utilizing a comprehensive panel comprising 29 immune-related features. These features broadly encompassed various immune cell types, functions, and related pathways. This detailed analysis was successfully executed using the “limma”, “GSVA” and “GSEABase” packages within the R software environment. Notably, a higher immune score derived from this analysis indicated active immune function within the samples. The samples were subsequently clustered into distinct groups based on their respective ssGSEA scores. This clustering was efficiently performed using the “sparcl” package. The robustness of immune score classification was verified through dimensionality reduction using t-Distributed Stochastic Neighbor Embedding (t-SNE) methodology. For a systematic characterization of TME, we leveraged the “limma” and “estimate” packages to compute four distinct parameters for each sample: stromal scores, immune scores, combined ESTIMATE scores, and tumor purity. These metrics provided a comprehensive assessment of the cellular composition and immune status within the TME. Visualization of TME heterogeneity was achieved through heatmap generation using the “pheatmap” package. Comparative analysis of immune subgroups was performed using violin plots, with statistical evaluation facilitated by the “reshape2” and “ggpubr” packages. To elucidate the underlying biological pathways associated with the immune landscape of LGG, we conducted KEGG pathway enrichment with stringent parameters: gene set size thresholds (with a minimum of 15 and a maximum of 500), statistical significance (*p* < 0.05), false discovery rate control (FDR < 0.25), and normalized enrichment score (NES > 1).

### 2.3. Evaluation of the LGG Microenvironment by CIBERSORT

CIBERSORT is an advanced deconvolution algorithm enabling precise characterization of immune cell composition through the analysis of gene expression data. In the present study, this computational methodology was employed to quantify the relative abundance of 22 immune cell populations infiltrating glioma specimens. The comprehensive immune cell repertoire analyzed encompassed various lymphocyte subsets (plasma cells, naive B cells, memory B cells, CD8+ T lymphocytes, naive CD4+ T lymphocytes, resting and activated memory CD4+ T cells, regulatory T cells [Tregs], follicular helper T cells, and gamma delta T cells); natural killer (NK) cell populations (both resting and activated states); monocytes; M0, M1, and M2 macrophages; antigen-presenting cells (resting and activated dendritic cells); granulocytes (eosinophils and neutrophils); and mast cells in both quiescent and activated states. This high-resolution immune landscape provided critical insights into the TME's cellular complexity and intercellular interactions within gliomas.

### 2.4. Discovery of Prognostic DEGs and Hub Gene Signatures in LGG

For identification of differentially expressed genes (DEGs) distinguishing the immunity_L and immunity_H subgroups, a stringent filtering protocol was implemented using the “limma” package in R. This analysis employed dual selection criteria: |log2 fold − change (FC)| > 0.585. Furthermore, IRGs were obtained by intersecting the DEGs with the immune-related genes. This intersection was visualized using the “venn” and “pheatmap” packages. To further elucidate the key players among these immune-related DEGs, we utilized Cytoscape software (Version 3.9.1) and the Cytohubba algorithm to identify hub genes. Then, we chose the Top 30 genes for in-depth analyses. Finally, we determined the core hub genes by identifying the common genes among the top-ranked lists based on various centrality measures, including Degree, MCC, MNC, EPC, and DMNC.

### 2.5. Establishment and Validation Protocol for a Prognostic Risk Assessment Model

To identify prognostic IRGs, we conducted Kaplan–Meier (KM) survival analysis, setting *p* < 0.05 to identify genes with statistically significant associations with survival outcomes. Following this initial screening, we employed two robust statistical methods, LASSO and multivariate COX regression analyses. For model training, we leveraged comprehensive survival data derived from LGG samples sourced from the TCGA and GTEx databases. To ensure the robustness of our model, the CGGA dataset, containing LGG samples with complete survival information, served as the validation set. Based on the median risk score derived from our established model, LGG patients were categorized into two groups: high-risk and low-risk. The risk score was computed using the formula: risk score = ∑*a*gene(*i*) × Expgene(*i*) where “*a*” represents the regression coefficient.

### 2.6. Independent Prognostic Evaluation and Construction of a Predictive Nomogram

To rigorously assess the efficacy of our model, we implemented time-dependent receiver operating characteristic (ROC) curves via the “survival,” “survminer,” and “timeROC” R packages. Next, we conducted both univariate and multivariate Cox regression analyses with detailed clinical information to assess the risk score, considering the risk model alongside clinical factors such as age, grade, and gender. Additionally, we developed a nomogram to predict the 1-year, 3-year, and 5-year overall survival (OS) rates for LGG patients. We followed the methods of Wang et al. [10] and Zhang et al. [11].

### 2.7. Risk Model–Driven Dissection of Immune Microenvironment Heterogeneity

To thoroughly examine and compare the differential abundance of various immune cell types between the two groups, we applied ssGSEA and ESTIMATE algorithms. These methodologies, which are rooted in gene expression data analysis, were specifically chosen for their ability to estimate the proportions of stromal and immune cells within malignant tumors. By utilizing these algorithms, we aimed to gain deeper insights into the variations in the immune microenvironment and the distinct patterns of cell infiltration that exist across the different risk groups. This comprehensive analysis would provide us with a clearer understanding of how the immune landscape differs between patients at varying levels of risk, potentially identifying key immune cell populations that contribute to prognosis and therapeutic responsiveness.

### 2.8. Exploration of CXCL10, ICAM1, IL18, ITGAL, SOCS3, and TLR3 Expression, Survival, and Immune Infiltration

To comprehensively study the expression and function of the six genes CXCL10, ICAM1, IL18, ITGAL, SOCS3, and TLR3, we performed extensive expression and survival analyses using data from TCGA and CGGA databases. These analyses allowed us to examine how expression levels of genes correlate with patient survival outcomes and other clinical parameters. Additionally, to gain a more nuanced understanding of the immune cell-specific expression of these genes, we additionally utilized the Tumor Immune Estimation Resource (TIMER) database. By leveraging this, we were able to elucidate the abundance and distribution of CXCL10, ICAM1, IL18, ITGAL, SOCS3, and TLR3 across various immune cell subsets. These detailed analyses are aimed at providing deeper insights into the potential functions and interactions within the complex tumor immune microenvironment, shedding light on their roles in shaping the immune landscape and influencing the progression and responsiveness to therapy of cancer.

### 2.9. Total RNA Extraction and Real-Time Quantitative Polymerase Chain Reaction (qPCR) Analysis

In our study, we collected 16 cases of LGG along with their corresponding paracancerous tissue samples to extract total RNA using M5 Total RNA Extraction Reagent (Catalog Number: MF034-01). The concentration and purity of total RNA in the samples were evaluated using Nanodrop 2000c with an OD260/OD280 ratio within the range of 1.8–2.2 indicating high purity. The process of reverse transcription was carried out in accordance with the manufacturer's guidelines, utilizing the Hifair III 1st Strand cDNA Synthesis SuperMix for qPCR (Catalog Number: 11141ES60, YEASEN, Shanghai, China), starting from 1.0 *μ*g of total RNA to synthesize complementary DNA (cDNA). Following that, qPCR analysis was conducted using the Hieff qPCR SYBR Green Master Mix (Catalog Number: 11201ES08, YEASEN, Shanghai, China). Differences in mRNA expression levels were calculated by the 2^−ΔCt^ method. Primer sequences are provided in Supporting Information 2: Table S1.

### 2.10. Western Blot Analysis

Tissue samples, each approximately the size of mung beans, were excised and used for the extraction of tissue proteins. The extraction was performed using RIPA buffer (Solarbio) that had been supplemented with protease inhibitors (SEVEN). Following the extraction, protein concentration in the samples was accurately quantified using a BCA protein quantification kit obtained from Solarbio. Subsequently, an equivalent amount of total protein, approximately 20 *μ*g, was then loaded onto 10% SDS-PAGE gels for the purpose of electrophoretic separation. The resulting resolved protein samples were meticulously transferred onto PVDF membranes (Millipore, Billerica, Massachusetts). Prior to antibody incubation, the PVDF membranes were blocked with 5% nonfat milk for 1 h. The membranes were incubated overnight at a temperature of 4°C with primary antibodies that were specific to CXCL10 (Nature Biosciences, dilution 1:1000); ICAM1 (Proteintech, dilution 1:2000), IL18 (ZENBIO, dilution 1:1000), ITGAL (ZENBIO, dilution 1:1000), SOCS3 (Proteintech, dilution 1:1000), TLR3 (Cloud-Clone Corp, dilution, 1:10000), GAPDH (Proteintech, 1:10000), and *β*-actin (Proteintech, 1:10000). The membranes underwent incubation with a secondary antibody diluted in blocking buffer the following day (typically 5% nonfat dry milk in TBST) for a duration of 1 h at ambient room temperature (approximately 22°C–25°C). This extended incubation period allowed sufficient time for antibody-antigen binding while maintaining optimal reaction conditions through constant gentle agitation on a rocking platform. Subsequently, the proteins were visualized using ECL, which allowed for the detection of the antibody-bound proteins. Finally, the grayscale values of the protein bands that were visualized on the membranes were quantitatively analyzed using ImageJ software, providing insights into the relative abundance of the proteins of interes.

### 2.11. Statistical Analysis

All data were analyzed by R studio and GraphPad Prism 8.0 software. Group comparisons were performed using Student's *t* test. Survival curves were generated through KM methodology implemented via R's “survminer” package, with statistical significance evaluated by log-rank testing. Complementary to these approaches, both univariate and multivariate Cox regression analyses were conducted. These analyses are aimed at identifying the factors that have a significant impact on OS. Univariate analysis allowed for examination of the association between each individual factor and OS, while the multivariate analysis took into account the potential confounding effects of multiple factors simultaneously. Intervariable relationships were quantified using Pearson's correlation coefficients, a parametric measure assessing linear association strength and directionality between paired variables. Statistical significance was defined as *p* < 0.05.

## 3. Results

### 3.1. ssGSEA and Immunocyte Infiltration Analysis

**Figure 1:**
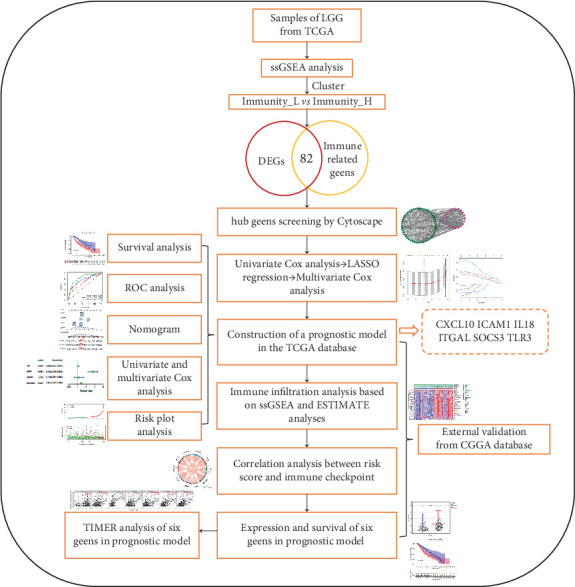
Workflow. Abbreviations: TCGA, The Cancer Genome Atlas; CGGA, Chinese Glioma Genome Atlas; ssGSEA, single-sample gene set enrichment analysis; ROC, receiver operator characteristic; DEGs, differentially expressed genes; LASSO, least absolute shrinkage and selection operator.

**Figure 2:**
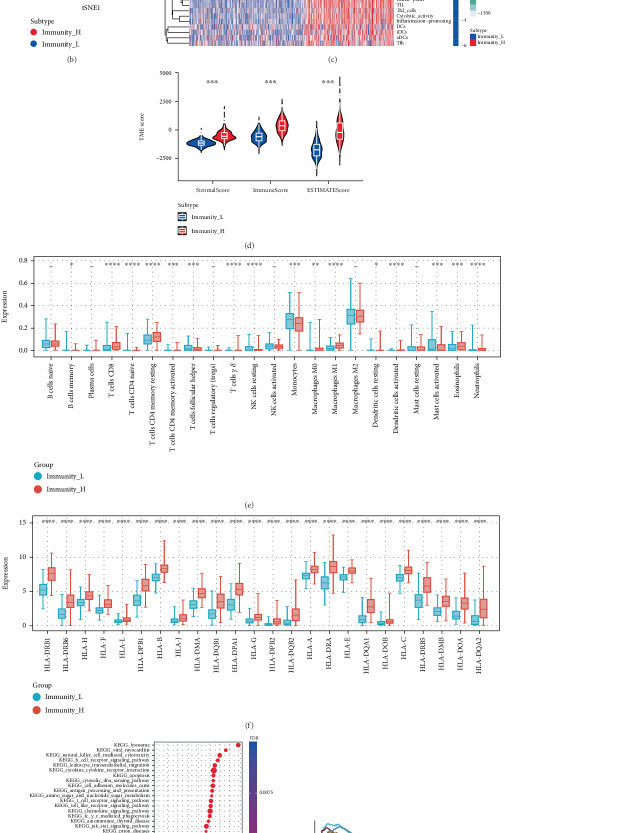
ssGSEA and CIBERSORT analysis of the immunity_L, immunity_H groups, and immunocyte infiltration (a) ssGSEA and cluster analysis of LGG. (b) tSNE analysis. (c) Heatmap of tumor purity, ESTIMATE score, immune score, and stromal score in the immunity_L and immunity_H groups. (d) Differential analysis of tumor microenvironment. (e) CIBERSORT analysis of the 22 immune cells in immunity_L and immunity_H groups. (f) Differences in human leukocyte antigen related genes between immunity_L and immunity_H groups. (g) KEGG signaling pathways enriched by GSEA enrichment analysis of immunity_L and immunity_H groups. (h) The immune-related signaling pathways.

The research workflow of the current investigation is systematically illustrated in Figure 1. The patient samples were categorized into two distinct subgroups designated as immunity_L and immunity_H cohorts, as visually represented in Figure 2a. The application of t-SNE analysis revealed that the immune score effectively discriminated patients belonging to the low immunity group from those in the high immunity group (Figure 2b). Comparative analysis of TME characteristics revealed substantial intergroup differences through heatmap visualization (Figure 2c). Specifically, the immunity_H group exhibited marked elevation in stromal scores (reflecting extracellular matrix components), immune scores (indicating immune cell infiltration), and composite ESTIMATE scores, concomitant with reduced tumor purity compared to the immunity_L cohort. Quantitative validation of these observations confirmed statistically significant enhancements in immune cell scores (*p* < 0.001), stromal cell scores (*p* < 0.001) and ESTIMATE scores (*p* < 0.001) in the high-immunity group (Figure 2d). To further characterize immune infiltration patterns, the CIBERSORT deconvolution algorithm was employed (Figure 2e). Bar plot analysis demonstrated significant elevation in immunity_H samples for multiple effector immune subsets including T cells CD8 (*p* < 0.001), T cells CD4 memory resting (*p* < 0.001), T cells CD4 memory activated (*p* < 0.001), T cells gamma delta (*p* < 0.001), macrophages M0 (*p* < 0.01), macrophages M1 (*p* < 0.001), dendritic cells activated (*p* < 0.001), Eosinophils (*p* < 0.001), neutrophils (*p* < 0.001), and dendritic cells resting (*p* = 0.02). By contrast, B cells memory (*p* = 0.04), T cells CD4 naive (*p* < 0.001), T cells follicular helper (*p* < 0.001), NK cells resting (*p* < 0.001), monocytes (*p* < 0.001), and mast cells activated (*p* < 0.001) showed reduced infiltration in the high-immunity group. Furthermore, transcriptomic profiling of human leukocyte antigen (HLA)-related genes revealed universal upregulation (p <0.001) of all 24 analyzed HLA molecules in the immunity_H cohort (Figure 2f). Pathway enrichment analysis through GSEA identified significant activation (Figure 2g) of immune-related KEGG pathways in high-immunity patients, including antigen processing and presentation, B cell receptor signaling pathway, cytokine cytokine receptor interaction, leukocyte transendothelial migration, NK cell mediated cytotoxicity, and T cell receptor signaling pathway, as detailed in Figure 2h.

### 3.2. Identification of DEGs and Hub Genes

**Figure 3:**
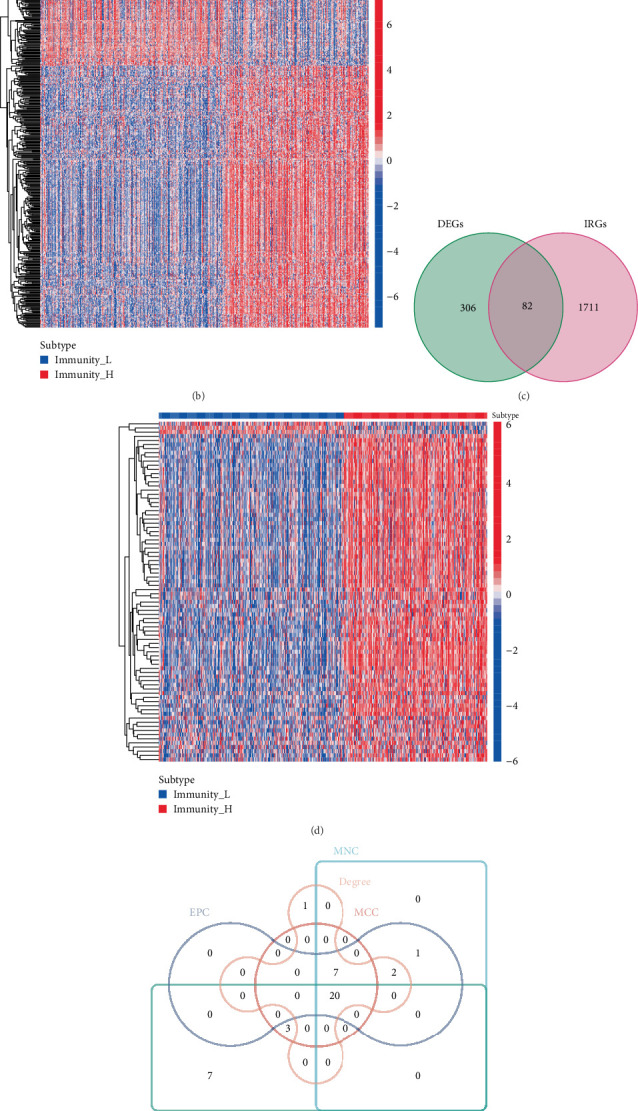
Screening of hub genes. (a) DEGs shown by a volcano plot with |log 2FC| ≥ 0.585. (b) Heatmap of differentially expressed genes. (c) Intersection between DEGs and IRGs displayed by the Venn diagram. (d) Heatmap of IRGs. (e) Intersection of Degree, MCC, MNC, EPC and DMNC of top 30 by Cytoscape software. (f) 20 hub genes displayed by Cytoscape.

Total 388 DEGs were screened, among which 299 were found to be upregulated and 89 were downregulated (Figure 3a). A heatmap of these DEGs is presented in Figure 3b. According to the Venn analysis, 82 genes were obtained from the intersection of the 388 DEGs and 1793 IRGs (Supporting Information 3: Table S2) were selected for further analysis (Figure 3c). The heatmap of 82 core genes was displayed in Figure 3d. For the visualization of core genes, Cytoscape software was utilized. CytoHubba was used to detect the most tumor-related core genes. Various algorithms, including Degree, MCC, MNC, EPC, and DMNC were applied to obtain the intersection of the Top 30 tumor-related genes. Finally, 20 hub genes were identified (Figure 3e,f).

### 3.3. Construction of IRG Model

**Figure 4:**
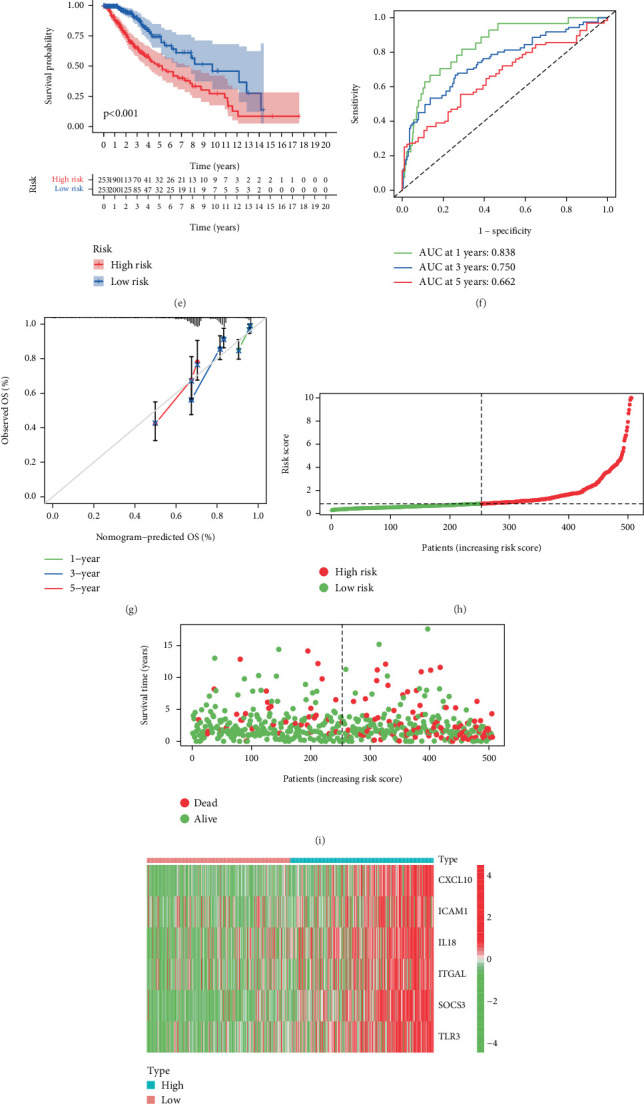
Construction of six genes signature and validation of the prognostic in the TCGA dataset. (a) Univariate Cox regression analysis of OS based on hub genes. (b, c) Construction and validation of IRGs signature. (d) Multivariate Cox regression analysis of six candidate genes. (e) Overall survival analysis of LGG patient risk score. (f, g) ROC and validation curves of 1-, 3-, and 5-year OS. (h–j) Distribution of risk score, survival time, and gene expression in LGG patients in the TCGA dataset. (k, l) Univariate and multivariate Cox regression analysis of age, gender, grade, and risk in the TCGA dataset. (m) The nomogram for predicting 1-, 3-, and 5-year survival outcomes of LGG patients integrating prognostic markers including grade, gender, age, and risk. (n) ROC of 1-, 3-, and 5-year nomogram. (o) Calibration of nomogram−predicted OS and observed OS.

Following initial identification of prognostic markers, rigorous statistical filtration was implemented through univariate Cox regression modeling to evaluate survival-associated genetic factors. This analytical phase identified 11 genes demonstrating statistically significant prognostic relevance (*p* < 0.001, Figure 4a). To address potential multicollinearity and optimize predictive accuracy, these preselected genetic candidates underwent dimensional reduction via LASSO regression (Figure 4b,c). Following the LASSO Cox regression analysis, eight genes were chosen. Subsequently, through multivariate Cox proportional hazards regression analysis, an IRG signature comprising six genes was identified and utilized to construct a prognostic characteristics: CXCL10, ICAM1, IL18, ITGAL, SOCS3, and TLR3 (Figure 4d). The risk score was computed following the mathematical formula:

 $$\begin{array}{c} \text{Risk Score}=\left(0.2518\times \text{expression of CXCL}10\right)+\left(-0.3294\times \text{expression of ICAM}1\right)+\left(0.3627\times \text{expression of IL}18\right)+\left(-0.5094\times \text{expression of ITGAL}\right)+\left(0.3185\times \text{expression of SOCS}3\right)+\left(0.6287\times \text{expression of TLR}3\right). \end{array}$$

Within the TCGA cohort, LGG patients underwent risk stratification through median risk score, creating distinct prognostic categories: low risk and high risk subgroups. KM survival exposed a pronounced divergence in clinical outcomes, with the high-risk cohort exhibiting markedly inferior survival probabilities (*p* < 0.001; Figure 4e). Temporal predictive performance evaluation through ROC analysis yielded area under curve (AUC) of 0.838 (1 year), 0.750 (3 years), and 0.662 (5 years) for OS prediction (Figure 4f). The calibration assessment curves demonstrated strong concordance between model-predicted survival probabilities and the actual survival rates across all evaluated timepoints (Figure 4g). Prediction curve and scatter plot illustrated each patient's risk score and survival status, with a higher concentration of deaths observed in high risk group (Figure 4h,i). Transcriptional patterns of the six-gene signature were systematically displayed through hierarchical clustering heatmap analysis (Figure 4j). Univariate cox proportional hazards regression identified advanced age (HR = 1.056; 95% CI = 1.042–1.071), higher tumor grade (HR = 3.426; 95% CI = 2.318–5.064) and elevated risk score (HR = 1.361; 95% CI = 1.274–1.453) as independent mortality predictors (all *p* < 0.001; Figure 4k). Multivariate adjustment preserved these associations, confirming the risk model's independent prognostic capacity (Figure 4l). Additionally, a nomograph shows the role of age, gender, grade, and risk in predicting OS (Figure 4m). The model demonstrated robust discriminative performance with time-specific AUCs of 0.882 (1-year), 0.843 (3-year), and 0.770 (5-year) (Figure 4n). Calibration curve analysis of the nomograph confirmed excellent alignment between predicted and observed survival outcomes (Figure 4o).

### 3.4. Validation of IRGs Model

**Figure 5:**
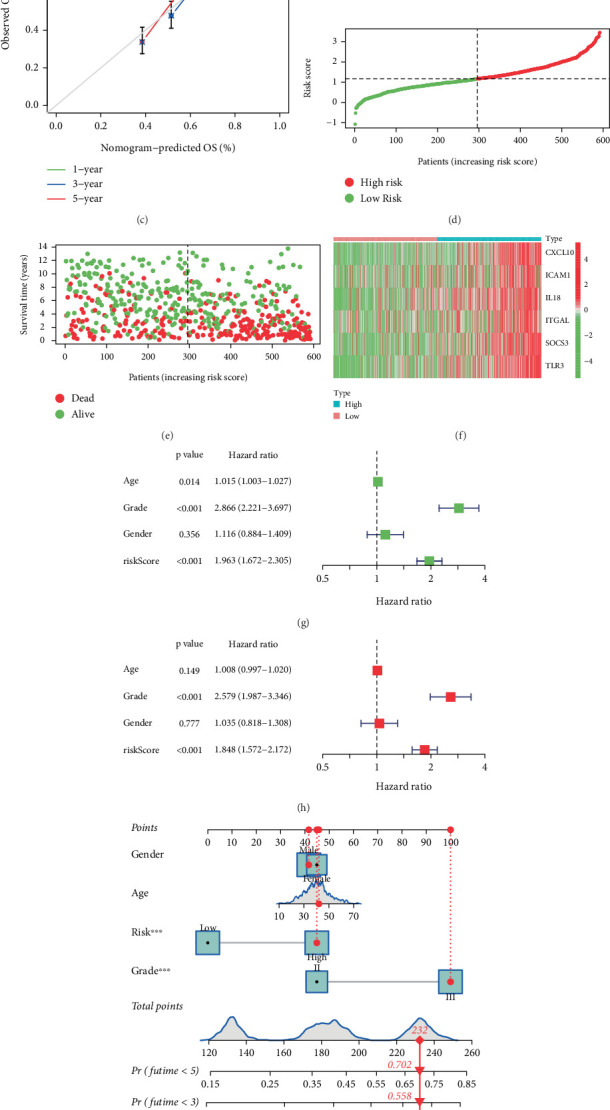
Validation of the prognostic in the CGGA dataset. (a) Overall survival analysis of LGG patient risk score. (b, c) ROC and validation curves of 1-, 3-, and 5-year OS. (d–f) Distribution of risk score, survival time, and gene expression in LGG patients in the CGGA dataset. (g, h) Univariate and multivariate Cox regression analysis of age, gender, grade, and risk in TCGA dataset. (i) The nomogram for predicting 1-, 3-, and 5-year survival outcomes of LGG patients integrating prognostic markers including grade, gender, age, and risk. (j) ROC of 1-, 3-, and 5-year nomogram. (k) Calibration of nomogram–predicted OS and observed OS.

We replicated the analysis in the CGGA dataset for validation. Comparative survival analysis revealed pronounced prognostic disparity between risk-stratified cohorts, with high-risk subgroup demonstrating poorer prognosis (*p* < 0.001; Figure 5a). Temporal predictive accuracy evaluation via ROC analysis yielded AUC values of 0.640 (1-year), 0.667 (3-year), and 0.672 (5-year) for OS prediction (Figure 5b). Survival rates predicted by the IRGs signature at 1-, 3-, and 5-year closely aligned with actual survival rates (Figure 5c). Prognostic curve, scatter plot, and expression levels were visualized using a heatmap (Figures 5d, 5e, and 5f). Prognostic factor evaluation through Cox proportional hazards regression modeling incorporated clinical–pathological variables (age, gender, and tumor grade) alongside risk stratification. Both univariate (Figure 5g) and multivariate (Figure 5h) analyses identified advanced tumor grade and elevated risk score as independent predictors of adverse outcomes. The nomograph was shown in Figure 5i. Model validation demonstrated robust discriminative capacity with AUCs of 0.706 (1-year), 0.743 (3-year), and 0.738 (5-year) for survival prediction (Figure 5j,k), underscoring the clinical utility of the IRG-based prognostic framework.

### 3.5. Immune Infiltration Analysis of IRG Model

**Figure 6:**
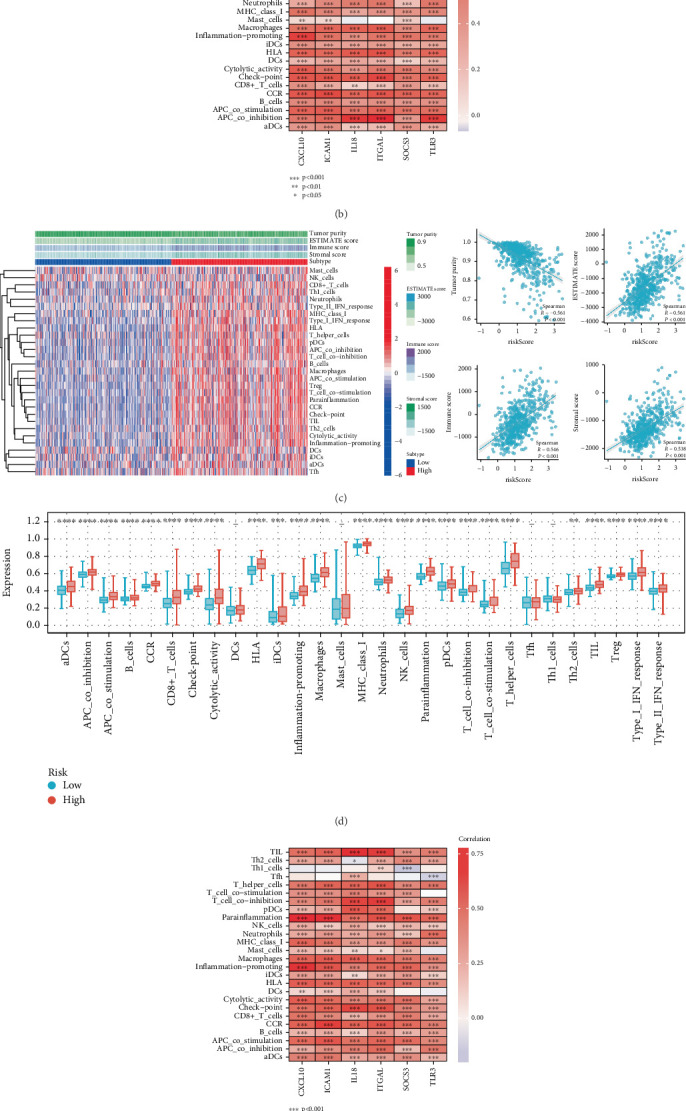
Immune infiltration analysis of IRGs based on ssGSEA algorithm. (a) The distribution of tumor-infiltrating immune cells in the TCGA database based on the ssGSEA algorithm. (b) The correlation between IRGs and immune cells in the TCGA dataset. (c) Tumor immune microenvironment scores for low-risk and high-risk populations based on ssGSEA and ESTIMATE analyses in the TCGA database. (d) The distribution of tumor-infiltrating immune cells in the CGGA dataset based on the ssGSEA algorithm. (e) The correlation between IRGs and immune cells in the CGGA dataset. (f) Tumor immune microenvironment scores for low-risk and high-risk populations based on ssGSEA and ESTIMATE analyses in the CGGA dataset.

The immune landscape within the TME was algorithmically quantified using the ssGSEA algorithm. The findings revealed, in the TCGA dataset, that nearly all immune cell types exhibited heightened expression in the high-risk subgroup, with marked elevations in B cells, CD8+ T cells, DCs, neutrophils, macrophages, and Tregs. Comparative analysis confirmed a statistically significant enhancement in overall immune infiltration scores within the high-risk cohort relative to low-risk counterparts (*p* < 0.001, Figure 6a). Subsequently, we conducted an in-depth analysis of the correlation between six genes and immunity. The results demonstrated a strong correlation between the six genes and immunity (Figure 6b). Quantitative characterization of TME components revealed significantly elevated stromal score, immune score, and ESTIMATE score within the high-risk cohort (*p* < 0.001, Figure 6c). These findings demonstrated cross-institutional reproducibility through concordant observations in the CCGA validation cohort (Figures 6d, 6e, and 6f).

### 3.6. Correlation Analysis Between Risk Model and Immune Checkpoint

**Figure 7:**
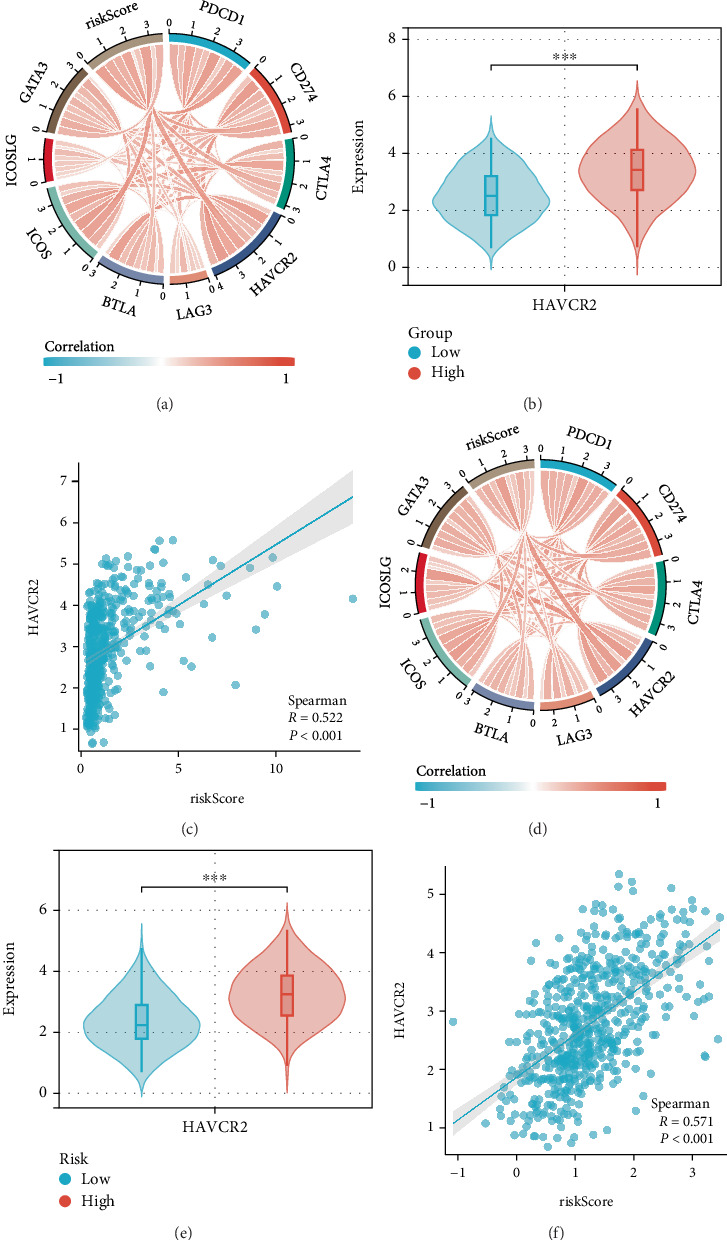
Correlation analysis between risk score and immune checkpoints. (a) Correlation chord diagram between risk score and immune checkpoint in the TCGA dataset. (b) Expression of HAVCR2 in low-risk and high-risk groups in the TCGA dataset. (c) Correlation analysis between the risk score and HAVCR2 in the TCGA dataset. (d) Correlation chord diagram between risk score and immune checkpoint in the CGGA dataset. (e) Expression of HAVCR2 in low-risk and high-risk groups in the CGGA dataset. (f) Correlation analysis between the risk score and HAVCR2 in the CGGA dataset.

Next, we systematically evaluated the association between immune checkpoint molecules and risk score that have been determined to serve as actionable targets for glioma intervention strategies, with the aim of assessing whether our model could predict the immunotherapeutic response in LGG. These immune checkpoints included PD-1 (PDCD1), PD-L1 (CD274), CTLA-4, TIM-3 (HAVCR2), LAG3, BTLA, ICOS, ICOSLG, and GATA3. Our results indicated a close relationship between risk score and immune checkpoint genes in TCGA and CGGA datasets (Figure 7a,d). Specifically, the risk score exhibited moderate-to-strong positive correlations with PD-1 (PDCD1) expression, yielding Pearson's correlation coefficients of 0.563 and 0.382 (Supporting Information 1: Figure S1a,b); PD-L1 (CD274) was 0.482 and 0.475 (Supporting Information 1: Figure S1c,d); CTLA-4 was 0.448 and 0.334 (Supporting Information 1: Figure S1e,f); LAG3 was 0.132 and 0.236 (Supporting Information 1: Figure S1g,h); BTLA was 0.310 and 0.304 (Supporting Information 1: Figure S1i,j); ICOS was 0.544 and 0.448 (Supporting Information 1: Figure S1k,l); ICOSLG was 0.217 and 0.331 (Supporting Information 1: Figure S1m,n); GATA3 was 0.406 and 0.253 (Supporting Information 1: Figure S1o,p) in TCGA and CGGA datasets. Detailed information between risk score and immune checkpoint expression profiles is provided in the Supporting Information section. Among them, transcriptomic analysis identified TIM-3 (HAVCR2) as demonstrating marked overexpression in high-risk patient group (Figure 7b,e). Bivariate correlation analysis using Pearson's method revealed significant positive associations between risk stratification and TIM-3 expression levels in both discovery (TCGA: Cor = 0.522, *p* < 0.001; Figure 7c) and validation (CGGA: Cor = 0.571, *p* < 0.001; Figure 7f).

### 3.7. Expression Levels and Survival Outcomes of Six IRG From Public Databases

**Figure 8:**
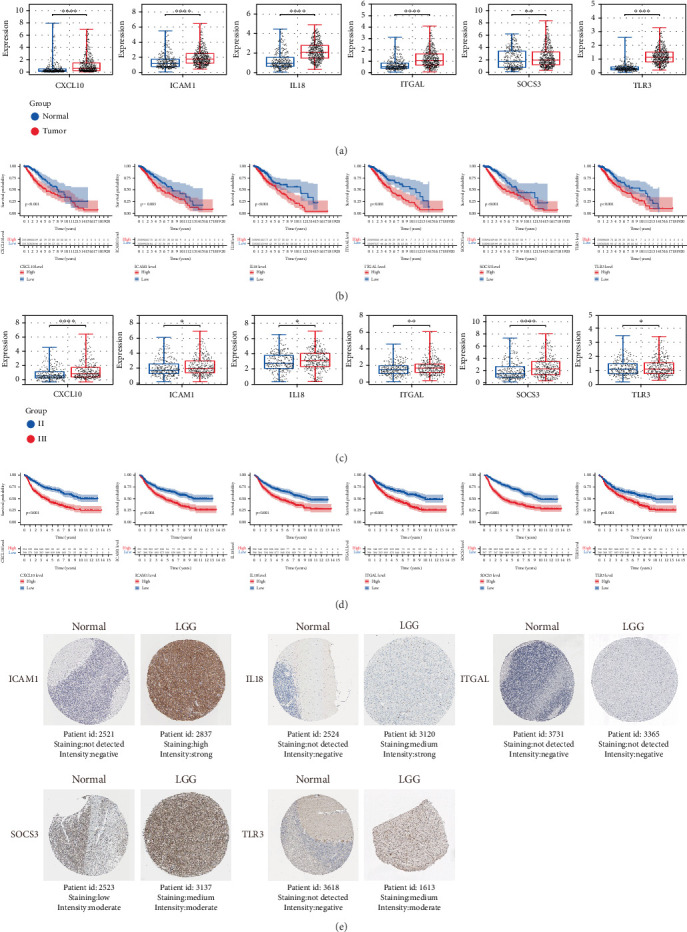
Expression and prognostic analysis of the six genes in the TCGA and CGGA datasets. (a, b) Expression and survival analysis of CXCL10, ICAM1, IL18, ITGAL, SOCS3, and TLR3 in the TCGA dataset. (c, d) Expression and survival analysis of CXCL10, ICAM1, IL18, ITGAL, SOCS3, and TLR3 in the CGGA dataset. (e) Protein expression of ICAM1, IL18, ITGAL, SOCS3, and TLR3 from the HPA website.

The expression and survival of CXCL10, ICAM1, IL18, ITGAL, SOCS3, and TLR3 were analyzed separately. Our findings found that the expressions of six genes were higher in tumors or gliomas with higher grades (Figure 8a,c). Furthermore, survival analysis demonstrated significant prognostic associations for the investigated gene signature, with elevated transcriptional levels correlating with inferior clinical outcomes in both TCGA and CGGA cohorts (Figure 8b,d). We searched the immunohistochemical results of these six factors from the Human Protein Atlas website and found that ICMA1, IL18, SOCS3, and TLR3 were expressed higher in LGG compared to normal tissues. However, ITGAL was not detected in either LGG or normal tissues. Immunohistochemical results for CXCL10 in both LGG and normal tissues were not retrieved from the database (Figure 8e).

### 3.8. qPCR and Western Blot Analysis of the Six IRGs

**Figure 9:**
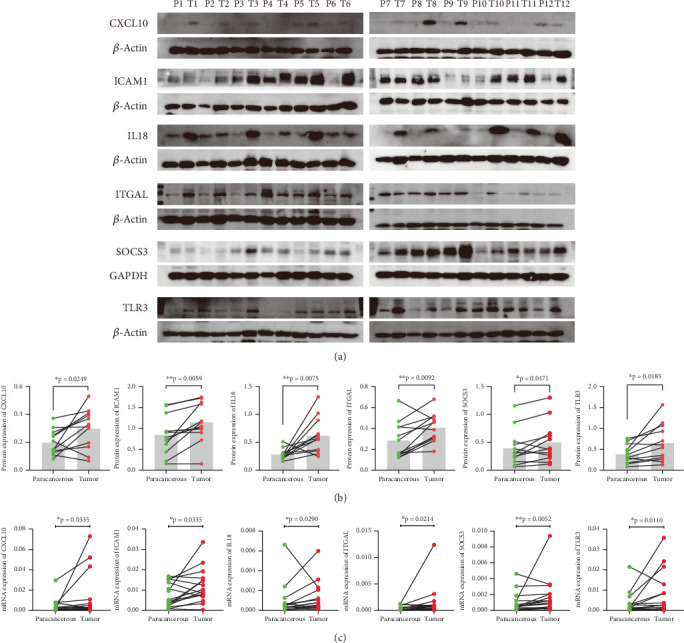
(a) Western blot analysis of paracancerous and tumor tissues from 12 patients with LGG in our center. (b) Quantitative results of western blot. (c) The qPCR analysis of the six genes signature.

We are curious about the actual RNA and protein expression levels of these factors in patients. Thus, we collected tumor tissues and their corresponding paracancerous tissues from 16 patients with LGG for qPCR detection, and another 12 pairs of tumor and paracancerous tissues were subjected to western blot to detect protein expression levels. Consistent with the results from public databases, we obtained similar findings in our samples for both RNA and protein expression levels. Specifically, the expressions of CXCL10, ICMA1, IL18, ITGAL, SOCS3, and TLR3 were higher in tumor tissues compared to their corresponding paracancerous tissues (Figures 9a, 9b, and 9c).

### 3.9. TIMER Analysis of the Six IRGs

**Figure 10:**
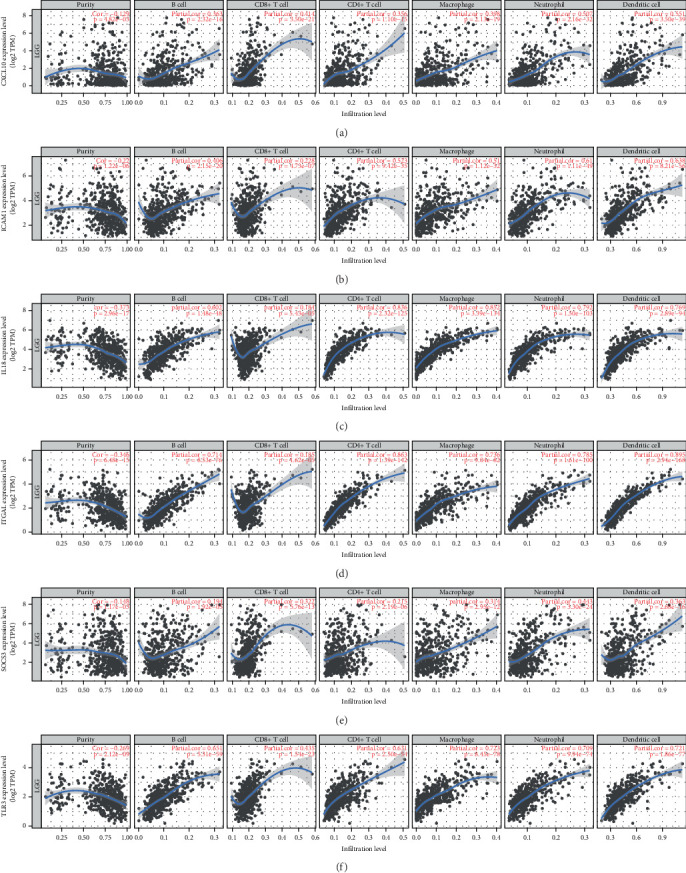
Immune infiltration analysis of IRGs using TIMER. (a) Relationship between the expression level of CXCL10, (b) ICAM1, (c) IL18, (d) ITGAL, (e) SOCS3, (f) TLR3 and purity, B cell, CD8+ T cell, CD4+ T cell, macrophage, neutrophil, and dendritic cell.

Based on the TIMER analysis, the expressions of CXCL10, ICAM1, IL18, ITGAL, SOCS3, and TLR3 in LGG patients were inversely correlated with tumor purity and positively correlated with various immune cell types, including B Cells, CD8+ T cells, CD4 + T cells, macrophages, neutrophils, and dendritic cells. Specifically, CXCL10 exhibited positive correlations with B cells (Cor = 0.363), CD8+ T cells (Cor = 0.414), CD4+ T cells (Cor = 0.356), macrophages (Cor = 0.398), neutrophils (Cor = 0.507), and dendritic cells (Cor = 0.551) (Figure 10a). ICAM1 demonstrated positive correlations with B cells (Cor = 0.406), CD4+ T cells (Cor = 0.523), macrophages (Cor = 0.51), neutrophils (Cor = 0.61), and dendritic cells (Cor = 0.638) (Figure 10b). IL18 showed positive correlations with B cells (Cor = 0.602), CD4+ T cells (Cor = 0.836), macrophages (Cor = 0.852), neutrophils (Cor = 0.792), and dendritic cells (Cor = 0.769) (Figure 10c). ITGAL displayed positive correlations with B cells (Cor = 0.714), CD4+ T cells (Cor = 0.863), macrophages (Cor = 0.736), neutrophils (Cor = 0.785), and dendritic cells (Cor = 0.895) (Figure 10d). SOCS3 exhibited positive correlations with CD8+ T cells (Cor = 0.322), macrophages (Cor = 0.314), neutrophils (Cor = 0.443), and dendritic cells (Cor = 0.363) (Figure 10e). TLR3 positively related to B cells (Cor = 0.651), CD8+ T cells (Cor = 0.435), CD4+ T cells (Cor = 0.631), macrophages (Cor = 0.723), neutrophils (Cor = 0.709), and dendritic Cells (Cor = 0.721) (Figure 10f).

## 4. Discussion

In 2020, 251,329 patients died of brain tumors, accounting for 2.5% of global cancer-related deaths [12]. Glioma stands as the most prevalent form of primary malignant brain tumor, accounting for about 75% of such cases in adult patients [13]. LGG has a highly variable clinical behavior, and its survival ranges from 1 to 15 years, which cannot be adequately predicted by histologic classification. Furthermore, owing to their highly aggressive characteristics, achieving complete neurosurgical resection remains unfeasible, often leaving residual tumor tissue that can subsequently lead to recurrence and malignant progression. Notably, while some gliomas remain indolent, others rapidly evolve into glioblastoma (WHO Grade IV glioma) within a few months. In recent years, despite the increasing utilization of gene classification to guide clinical decision-making, such as IDH, TP53, and ATRX mutations, 1p/19q codeletion, which are recognized as relevant markers of LGG [2], significant individual variations among LGG patients persis. Even with the inclusion of additional molecular typing, the clinical outcome of these patients remains incompletely predictable. Consequently, there is a pressing need to identify novel molecular biomarkers for LGG, which could offer fresh insights into the diagnosis, treatment, and prognosis of LGG, ultimately benefiting LGG patients through our discoveries.

Previous studies have established IRG signatures as independent prognostic factors in glioblastoma multiforme (GBM) [14, 15], highlighting the potential of these signatures to reflect immune status and local immune responses. However, the prognostic interplay between immune microenvironment dynamics and risk signatures in LGG remains to be fully characterized. In our current study, 82 IRGs were identified. Subsequent algorithmic selection via LASSO regression followed by multivariate Cox proportional hazards regression distilled a six-gene prognostic signature comprising CXCL10, ICAM1, IL18, ITGAL, SOCS3, and TLR3, with all coefficients demonstrating statistical significance. By utilizing the median risk score as a cutoff, the IRG signature successfully stratified LGG patients into two subgroups. Subsequent analyses, including KM curves, ROC curves, and risk plots, confirmed the robustness of six-gene risk signature in primary LGG within both TCGA and CGGA datasets. Importantly, both tumor grade and risk stratification group demonstrated independent prognostic significance for OS in LGG patients through both univariate and multivariate Cox regression analyses. Furthermore, our signature maintained robust temporal stability across OS predictions, as validated through rigorous multicohort analyses encompassing both internal (TCGA) and external (CGGA) datasets.

In the present investigation, six IRGs were algorithmically derived through systematic bioinformatics analysis. Emerging evidence from functional genomics studies elucidates the multifaceted roles of these genes in orchestrating and regulating the glioma immune response. CXCL10 (C-X-C motif chemokine ligand 10) operates as a potent proinflammatory mediator that orchestrates diverse biological activities encompassing chemotaxis, differentiation, and activation of peripheral immune cells, as well as the regulation of cell growth, apoptosis, and modulation of angiostatic effects, with recent studies highlighting its pivotal role in glioma immunology. In vitro experimental models of glioma cell-intrinsic mechanisms revealed that IDH mutation may inhibit recruitment of CD8 + T cells via CXCL10 downregulation [16], while Sarah and his team observed that treatment with GSK343 modulated the innate immune response in GBM by elevating CXCL9, CXCL10, and CXCL11 expression levels, enhancing the migration of NK cells to tumor sites and consequently promoting NK cell-mediated tumor growth inhibition [17]. Furthermore, Mitsugu and his colleagues found that mouse DC1s displayed phenotypic characteristics consistent with mature dendritic cells and secreted elevated amounts of IL-12 and CXCL10, thereby indicating the pivotal involvement of CXCL10 in both the immunogenic and therapeutic efficacy of dendritic cell-based cancer immunotherapy strategies [18]. .ICAM1 (intercellular adhesion molecule 1), also designated as CD54, represents a cell surface glycoprotein encoded by the ICAM1 gene and is categorized within the immunoglobulin family. It is commonly expressed on endothelial cells and immune system cells, playing an important role in facilitating adhesion at sites of inflammation, controlling tumor progression and metastasis, and regulating the immune response [19]. Numerous studies have confirmed that ICAM1, as an oncogene, plays a key role in pathological processes of various types of tumors, including colorectal [20] and breast cancer [21]. Notably, the MSI/ICAM1 pathway is highly expressed in GBM cells, playing an important role in oncogenic resistance, particularly in enhancing tumor invasion, suggesting that MSI1/ICAM1 represents a promising therapeutic target for GBM [22]. Ding et al. elucidated that oncogenic mutant isocitrate dehydrogenase 1 (IDH1), a frequent genetic alteration in gliomas, enhances the phagocytic function of microglia/macrophages within the TME through transcriptional repression of ICAM1 expression [23]. Building on these findings, Ki-Chun et al. demonstrated a distinct ICAM1-related immunomodulatory axis in GBM. Their work revealed that ionizing radiation induces GBM cells to shed soluble ICAM1 (sICAM-1) into the tumor milieu, which stimulates the infiltration of macrophages, thereby enriching the TME with inflammatory macrophages [24]. IL18 (Interleukin 18) is a proinflammatory cytokine with significant functions, including the induction of angiogenesis and regulation of immune function. It is implicated in the progress of many inflammatory diseases, immune disorders, and tumors. Park's team speculated that IL18 contributes to the poor prognosis of triple-negative breast cancer patients by inducing immunosuppression through PD-1 expression on NK cells. IL-18, a pleiotropic proinflammatory cytokine with pleiotropic biological activities, serves as a critical regulator of angiogenic processes and orchestrates complex immune responses. Emerging evidence has mechanistically implicated this cytokine in the pathogenesis and progression of multiple pathological states, encompassing inflammatory disorders, immune dysregulation syndromes, and oncogenic malignancies. Of particular clinical relevance, Park's research group demonstrated that IL-18 contributes to the adverse prognostic outcomes in triple-negative breast cancer patients through a novel immunosuppressive mechanism involving PD-1 receptor upregulation on NK cells, thereby attenuating antitumor immunity [25]. Furthermore, IL-18 is highly expressed in the serum and tissues of glioma patients [26, 27], indicating its potential involvement in glioma pathogenesis. IL-18 binding protein (IL-18BP) may modulate the antitumor activity of IL-18 and influence the function of Tregs through interaction with IL-37 [26]. ITGAL (Integrin subunit alpha L) is a receptor for ICAM1, ICAM2, ICAM3, and ICAM4, and encodes lymphocyte function-related antigens. As a member of the Integrin family, ITGAL plays a significant role in tumor chemotaxis and immune responses [28]. ITGAL/CD11a demonstrates marked enrichment in glioma-associated microglia/macrophages (GAMs) derived from both murine models and human LGG specimens. This transmembrane protein modulates GAMs motility and CCL5 production in vitro, while concurrently functioning as a dual-purpose biomarker for disease monitoring and a promising therapeutic target for pharmacological modulation in this specific microglial population [29]. SOCS3 is a member of the suppressor of cytokine signaling (SOCS) family, functioning as a cytokine-inducible protein that inhibits cytokine signaling across multiple signaling pathways. SOCS3 may regulate tumor development through various physiological and pathological processes [30]. Dai' study revealed that high SOCS3 expression is associated with glioma and poor prognosis [31]. Braden's study demonstrated that myeloid cells lacking SOCS3 expression exhibit augmented and sustained production of proinflammatory M1-type cytokines upon exposure to glioma-derived conditioned media in vitro. This SOCS3 deficiency in macrophages was associated with a significant reduction in M2-polarized tumor-associated macrophage (TAM) infiltration into neoplastic tissues. Furthermore, the absence of SOCS3 in myeloid lineages resulted in a pronounced increase in cytotoxic CD8+ T lymphocyte tumor infiltration coupled with decreased presence of immunosuppressive Tregs within the TME [32]. .Toll-like receptor 3 (TLR3), a protein also identified as CD283, belongs to the TLR family of pattern recognition receptors that serve as essential sentinels in detecting pathogen-associated molecular patterns (PAMPs). This receptor complex constitutes a critical component of the innate immune system by mediating the initial recognition of microbial components and triggering subsequent inflammatory responses. A study has demonstrated that suppressed expression of TLR3 contributed to prolonged survival outcomes in LGG patients. TLR3 exerts its effects on tumors primarily by modulating the immune microenvironment of LGG [33]. Huang's study demonstrated that dual stimulation of TLR3/TLR9 pathways in myeloid cells of the microglia/macrophage lineage enhances glioma suppression efficacy [34]. .In our study, CXCL10, ICAM1, IL18, ITGAL, SOCS3, and TLR3 were all found to be highly expressed in LGG with poor prognosis. The results from TIMER analysis revealed that the above-mentioned six factors exhibited a strong correlation with B cells, CD8+ T cells, CD4+ T cells, macrophages, neutrophils, and dendritic cells. Furthermore, we analyzed the correlation between these six genes and immune function. Results indicated that these six genes were highly correlated with immune function based on the ssGSEA algorithm.

Chen et al. analyzed multiple sequencing datasets based on AML to assess immune infiltration in AML samples. Two immune-based AML subgroups, G1 and G2, were finally identified. G1 exhibited higher immune infiltration, more monocyte phenotype, and increased monocyte/macrophage ratio [35]. To characterize the tumor immune landscape in LGG patients, we implemented multidimensional computational analyses integrating ssGSEA and CIBERSORT algorithms. CIBERSORT analysis corroborated that LGG cells enriched via immune cells secretion [36]. A correlation heatmap generated from our analysis demonstrated differential distribution patterns between risk strata, where the high risk cohort exhibited significantly elevated immune, stromal and ESTIMATE scores. Conversely, tumor purity showed reciprocal decrease in the high-risk subset. These observations collectively imply that LGG patients in the high risk category manifest enhanced immune cell infiltration and more intricate immune activation profiles, suggesting a dynamic interplay between tumor progression and immune surveillance mechanisms.

Over the past few years, the realm of cancer treatment has witnessed significant advancements, particularly due to the groundbreaking success of immune checkpoint blockade immunotherapies. Immune checkpoint inhibitors (ICIs), a class of immunomodulatory agents that govern immune cell functionality, represent a cornerstone in contemporary cancer immunotherapy by serving as critical regulators of adaptive immune responses [37]. Consequently, the research focus on targeting immune checkpoint proteins has become one of the most active areas in cancer research. Notably, checkpoint inhibitors targeting the PD-1/PD-L1 or CTLA-4 have been approved for the treatment of various cancers. However, there are still a large number of patients who do not benefit from these treatments, prompting the exploration of novel immune checkpoint receptors [8]. Among checkpoint proteins under investigation, TIM3 has emerged as a critical target attracting intensive research focus. As a member of the TIM gene family, the transmembrane protein functions as a pivotal negative regulator of immune responses with documented expression on T cells, macrophages, Treg cells, NK cells, and tumor cells. The interaction between TIM3 and its ligand, Galectin-9 (LGALS9), triggers T-cell dysfunction characterized by impaired proliferative capacity and cytokine secretion deficiency, ultimately fostering tumor-associated immunosuppression and immune escape mechanisms [38, 39]. Our study revealed a robust association between our riskscore and CTLA4, PDCD1, CD274, ICOS, BTLA, and TIM3 in both TCGA and CGGA datasets. The riskscore had the strongest correlation with TIM3. These results suggest a close link between our model and immune cell infiltration as well as immune checkpoints. Our data support the hypothesis that the developed biomarker signature may constitute an innovative framework for stratifying patients and optimizing targeted immunotherapy regimens.

This study is subject to limitations. Firstly, although two datasets, TCGA and CGGA, were used for validation, the inclusion of multicenter data was inadequate. In future endeavors, we plan to incorporate more data from additional hospitals pertaining to glioma patients. Within the scope of this study, we have demonstrated the capacity of IRGs to predict outcomes accurately in LGG patients. Furthermore, our analysis pointing at immune cell infiltration revealed a close correlation between IRG features and immune cell infiltration within the LGG microenvironment. This finding offers a novel approach and strategy for the immunotherapy of LGG.

## 5. Conclusion

We developed a novel immune-related risk stratification model that established risk scores as an independent prognostic indicator for LGG. Subsequent analyses unveiled significant disparities in immune cell infiltration patterns among patient subgroups, suggesting that variations in tumor immune microenvironment may underlie poor clinical outcomes in specific cohorts. These findings collectively offer a promising biomarker framework to guide personalized therapeutic strategies for LGG management.

## Data Availability

The datasets generated and/or analyzed during the current study are presented in the main file. Additional data are available from the corresponding author on reasonable request.

## Supporting Information

Supporting Information 1 Figure S1: Correlation analysis between risk score and immune checkpoints. (a, b) Correlation chord diagram between risk score and PD-1 (PDCD1) in the TCGA and CGGA datasets. (c, d) Correlation chord diagram between risk score and PD-L1 (CD274) in the TCGA and CGGA datasets. (e, f) Correlation chord diagram between risk score and CTLA-4 in the TCGA and CGGA datasets. (g, h) Correlation chord diagram between risk score and LAG3 in the TCGA and CGGA datasets. (i, j) Correlation chord diagram between risk score and BTLA in the TCGA and CGGA datasets. (k, l) Correlation chord diagram between risk score and ICOS in the TCGA and CGGA datasets. (m, n) Correlation chord diagram between risk score and ICOSLG in the TCGA and CGGA datasets. (o, p) Correlation chord diagram between risk score and GATA3 in the TCGA and CGGA datasets.

Supporting Information 2 Table S1: Primer sequences for CXCL10, ICAM1, IL18, ITGAL, SOCS3, and TLR3.

Supporting Information 3 Table S2: 1793 immune-related genes obtained from the ImmPort website.
