# Supplementary figures and images for: Systematic Analysis of an Immune-Related Gene Signature for Predicting Prognosis and Immune Characteristics in Primary Lower Grade Glioma
Source: Biomed Res Int. 2025 Aug 12;2025:6180391. doi: 10.1155/bmri/6180391 (PMC12364596; doi:10.1155/bmri/6180391)

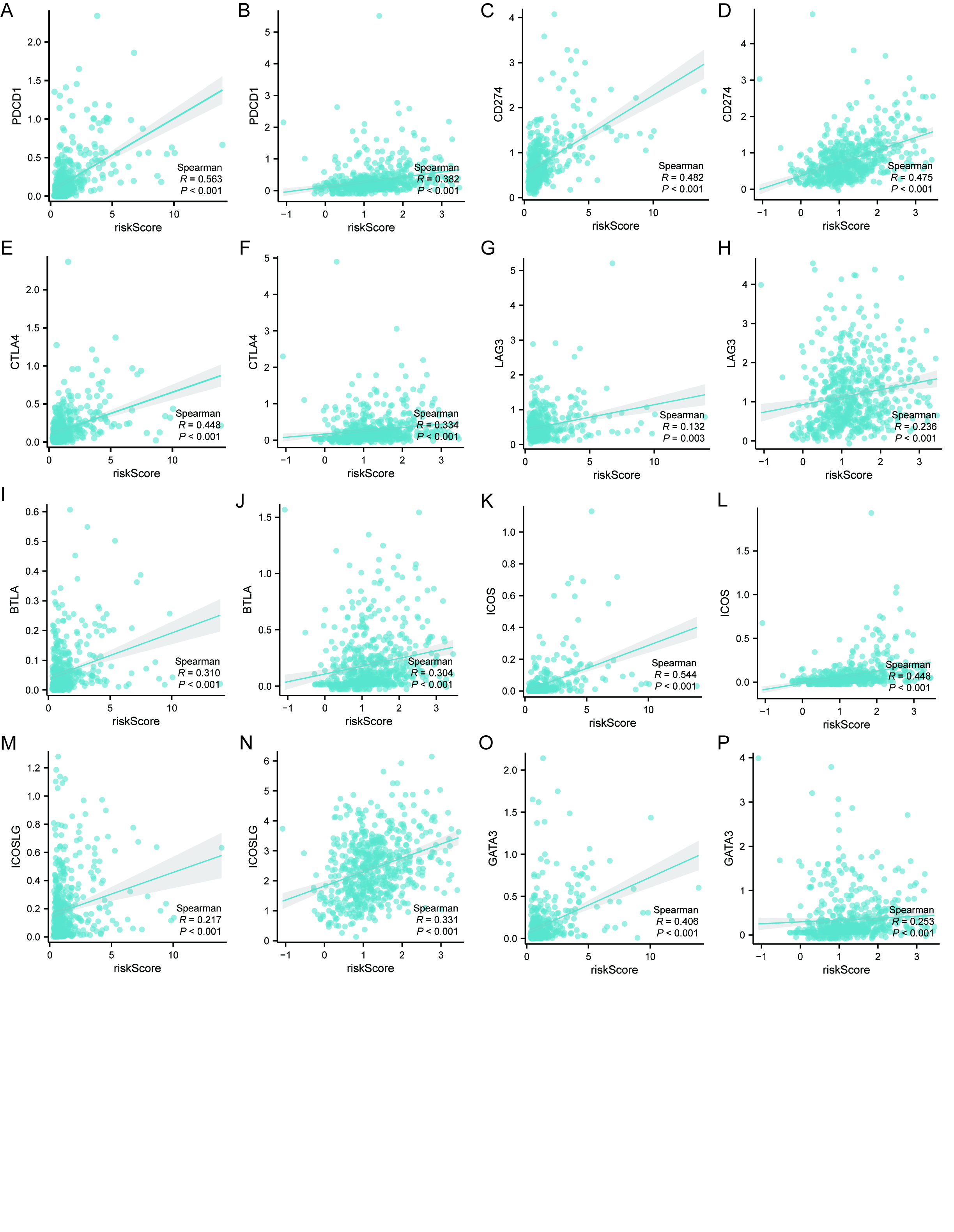

Supplement: Supporting Information 1 — Figure S1: Correlation analysis between risk score and immune checkpoints. (a, b) Correlation chord diagram between risk score and PD-1 (PDCD1) in the TCGA and CGGA datasets. (c, d) Correlation chord diagram between risk score and PD-L1 (CD274) in the TCGA and CGGA datasets. (e, f) Correlation chord diagram between risk score and CTLA-4 in the TCGA and CGGA datasets. (g, h) Correlation chord diagram between risk score and LAG3 in the TCGA and CGGA datasets. (i, j) Correlation chord diagram between risk score and BTLA in the TCGA and CGGA datasets. (k, l) Correlation chord diagram between risk score and ICOS in the TCGA and CGGA datasets. (m, n) Correlation chord diagram between risk score and ICOSLG in the TCGA and CGGA datasets. (o, p) Correlation chord diagram between risk score and GATA3 in the TCGA and CGGA datasets. [file 6180391.f1.zip › Supplemental Figure1S.tif]
